# Forensic Impact of the Omics Science Involved in the Wound: A Systematic Review

**DOI:** 10.3389/fmed.2021.786798

**Published:** 2022-01-06

**Authors:** Aurelia Collados Ros, Stefano Bacci, Aurelio Luna, Isabel Legaz

**Affiliations:** ^1^Department of Legal and Forensic Medicine, Biomedical Research Institute (IMIB), Regional Campus of International Excellence “Campus Mare Nostrum”, Faculty of Medicine, University of Murcia, Murcia, Spain; ^2^Department of Biology, Research Unit of Histology and Embriology, University of Florence, Florence, Italy

**Keywords:** human skin wounds, omics sciences, vital wounds, forensic sciences, age wound

## Abstract

**Background:** In forensic autopsies, examining the wounds is one of the most critical aspects to clarify the causal relationship between the cause of death and the wounds observed on the corpse. However, on many occasions, it is difficult to differentiate antemortem injuries from post-mortem injuries, mainly when they occur very close to the moment of death. At present, various studies try to find biomarkers and clarify the molecular mechanisms involved in a wound due to the high variability of conditions in which they occur, thus being one of the most challenging problems in forensic pathology. This review aimed to study the omics data to determine the main lines of investigation emerging in the diagnosis of vital injuries, time of appearance, estimation of the age and vitality of the wound, and its possible contributions to the forensic field.

**Methods:** A systematic review of the human wound concerning forensic science was carried out by following PRISMA guidelines.

**Results:** This study sheds light on the role of omics research during the process of wounding, identifying different cytokines and other inflammatory mediators, as well as cells involved in the specific stage of the wound healing process, show great use in estimating the age of a wound. On the other hand, the expression levels of skin enzymes, proteins, metal ions, and other biomarkers play an essential role in differentiating vital and post-mortem wounds. More recent studies have begun to analyze and quantify mRNA from different genes that encode proteins that participate in the inflammation phase of a wound and miRNAs related to various cellular processes.

**Conclusions:** This study sheds light on the role of research in the molecular characterization of vital wounds, heralding a promising future for molecular characterization of wounds in the field of forensic pathology, opening up an important new area of research.

**Systematic Review Registration:** URL: https://www.crd.york.ac.uk/prospero/#myprospero, Identifier: CRD42021286623.

## Introduction

Estimating the age and vitality of human skin wounds in the living and dead is essential in forensic practice (1, 2). Due to supravital reactions and minor morphological changes evident during this time, immunohistochemical parameters for age assessment and vitality for human skin wounds remain challenging (3–6).

Antemortem wounds elicit vital reactions that do not occur in post-mortem wounds, so the demonstration of a vital injury is sufficient to affirm that the injury occurred before death. On the other hand, vital reactions follow regular and time-dependent courses, which allows a reliable temporal classification of wound healing (7). Extravasations of red blood cells and hemoglobin to the wound were once considered a vital reaction indication, but considerable research have contradicted these findings, indicating that it cannot be utilized as a good marker in wound vitality diagnosis (8).

In this context, forensic molecular pathology includes applying omics sciences to investigate the genetic basis and the cause of death at the molecular biological level. Today many genomic investigations carry out an analysis of the genetic background (9–11), study the dynamics of gene expression (transcriptomics) is also playing an important role (12–14), as well as vital phenomena involving activated biological mediators and their degenerative products (proteomics) (15), and finally, the analysis of the different metabolites involved (metabolomics) (16–18).

Post-mortem biochemistry and experimental research propose the use of molecular biology techniques in the context of forensic pathology to detect functional changes involved in the death process, which cannot be detected morphologically (1, 19). In this context, different studies have shown that many cytokines, growth factors, and proteases are involved in the healing process of a wound, their study being practical to determine the vitality of the wound or its age in forensic medicine (20).

Besides, there are different approaches to assess the vitality of a lesion, from macromorphology to the level of mRNA through histology and protein. However, in the last 30 years, immunohistochemical techniques have been the method of choice to study wound vitality and age (21). However, elucidating the link between wounds and mortality causes utilizing specific vitality indicators with an adequate and consistent strategy remains a subject of controversy (6, 22).

The main objective of this systematic review was to compile the main lines of research on age and differentiation of vital and post-mortem human wounds that have emerged in recent years and their relationship with omics sciences, as well as the possible contributions or limitations in the field of forensic sciences.

## Systematic Review

The methods used for this systematic review (covering 1992 to July 2021) were developed by reference to the Preferred Reporting Items for Systematic Reviews and Meta-Analyses (PRISMA) statement (23) for studies published in accordance with the methods detailed in the Cochrane Handbook for Systematic Reviews of Interventions (24). Before commencement, the protocol for this systematic review was registered with the International Prospective Register of Systematic Reviews (PROSPERO, CRD42021286623).

### Inclusion Criteria

All studies exploring the vitality and the age of wounds in human forensic science in subjects aged 0–97 years old were included. The articles were chosen according to three main inclusion criteria: (i) studies of the age of vitality wounds, (ii) differentiation of vital and post-mortem wounds, and (iii) wounds of human origin.

### Search Strategy

Literature search strategies were developed in collaboration with a health sciences librarian using two scientific electronic databases (PubMed and Scopus) and keywords.

For the articles included in the review, the key characteristics of the studies were identified: topic discussed, first author, and year. The following keywords and subject heading terms were used: [[(wounds) OR (injuries)] AND (skin)] AND (forensic). The search in the two scientific electronic databases (PubMed and Scopus) was limited to articles published in English and studies conducted in humans. Two independent reviewers revised titles and abstracts and then full-text publications concerning the inclusion criteria. Study selection interrater agreement between the two reviewers was calculated as the proportion of favorable agreement (25).

### Data Extraction

Two independent testers retrieved duplicate data using Microsoft Excel. We checked and compared multiple reports from the same study and extracted them where specific data existed. For all studies that met the inclusion criteria, the following data were extracted: authors, year of publication, geographic location, study population, study design, sample size, age range, post-mortem interval, gender, type of wound, biomarker detected, and technique used.

### Risk of Bias Assessment

The risk of bias was assessed for each sample by comparison with the Cohort Research Checklist of the Critical Assessment Skills Program (CASP) (26). The following confounding variables within the CASP checklist were evaluated: sample size, age, post-mortem interval, gender, and analyses technique. Based on the CASP checklist, study output was graded as “bad,” “fair,” or “good.” The overall quality of the proof was rated as high, moderate, weak, or extremely low (27).

### Descriptive Studies

**Figure 1:**
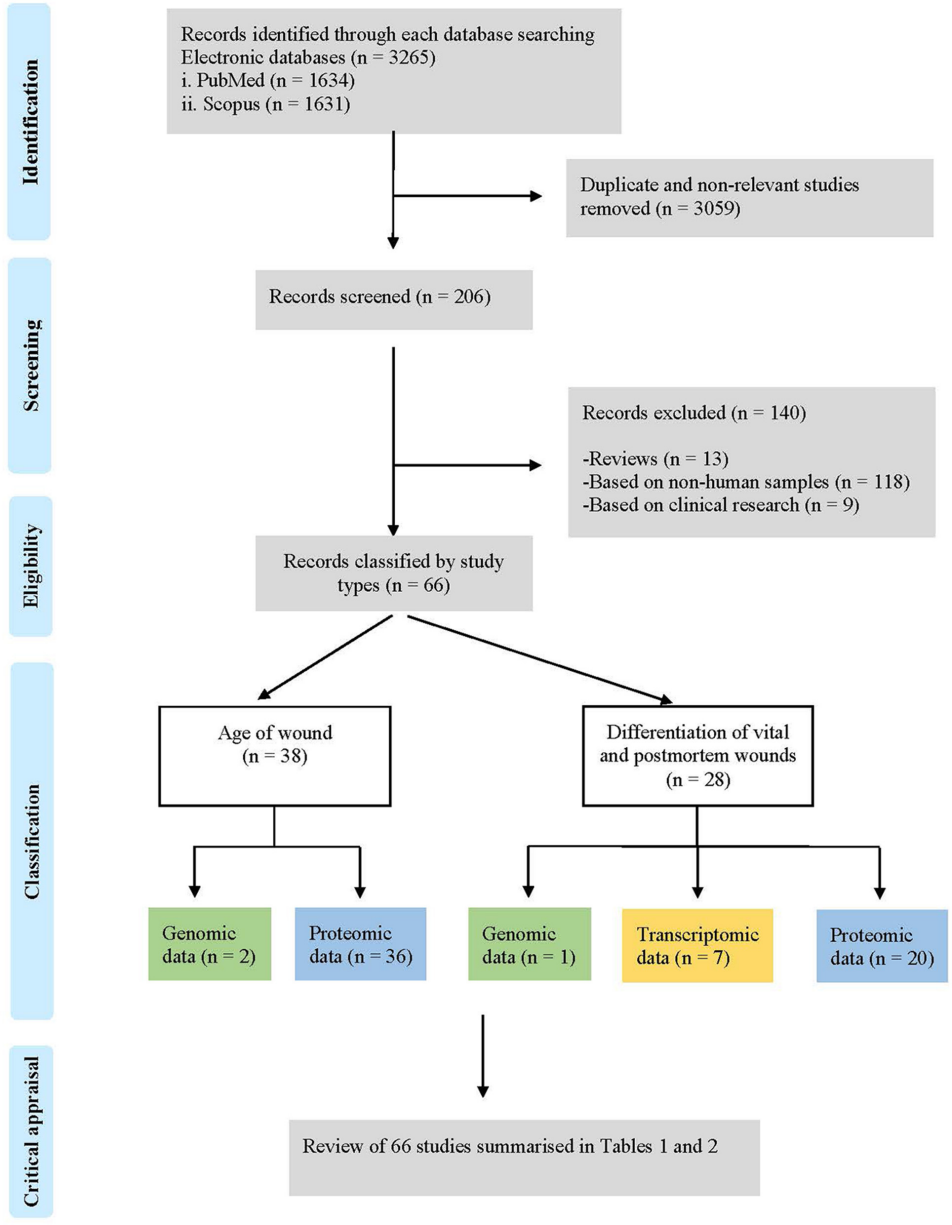
Flow chart of the literature search process and study selection according to PRISMA (Preferred reporting items for systematic reviews and meta-analysis) guidelines.

A total of 3.265 studies were identified in the two scientific electronic databases, PubMed (1.634) and Scopus (1.631) (Figure 1). A total of 3.059 duplicates and non-relevant studies were eliminated, and 206 studies were reviewed to assess their relevance. A total of 140 studies were excluded by these criteria: (i) reviews (*n* = 13); (ii) based on non-human samples (*n* = 118) and (iii) based on clinical research (*n* = 9).

**Figure 2:**
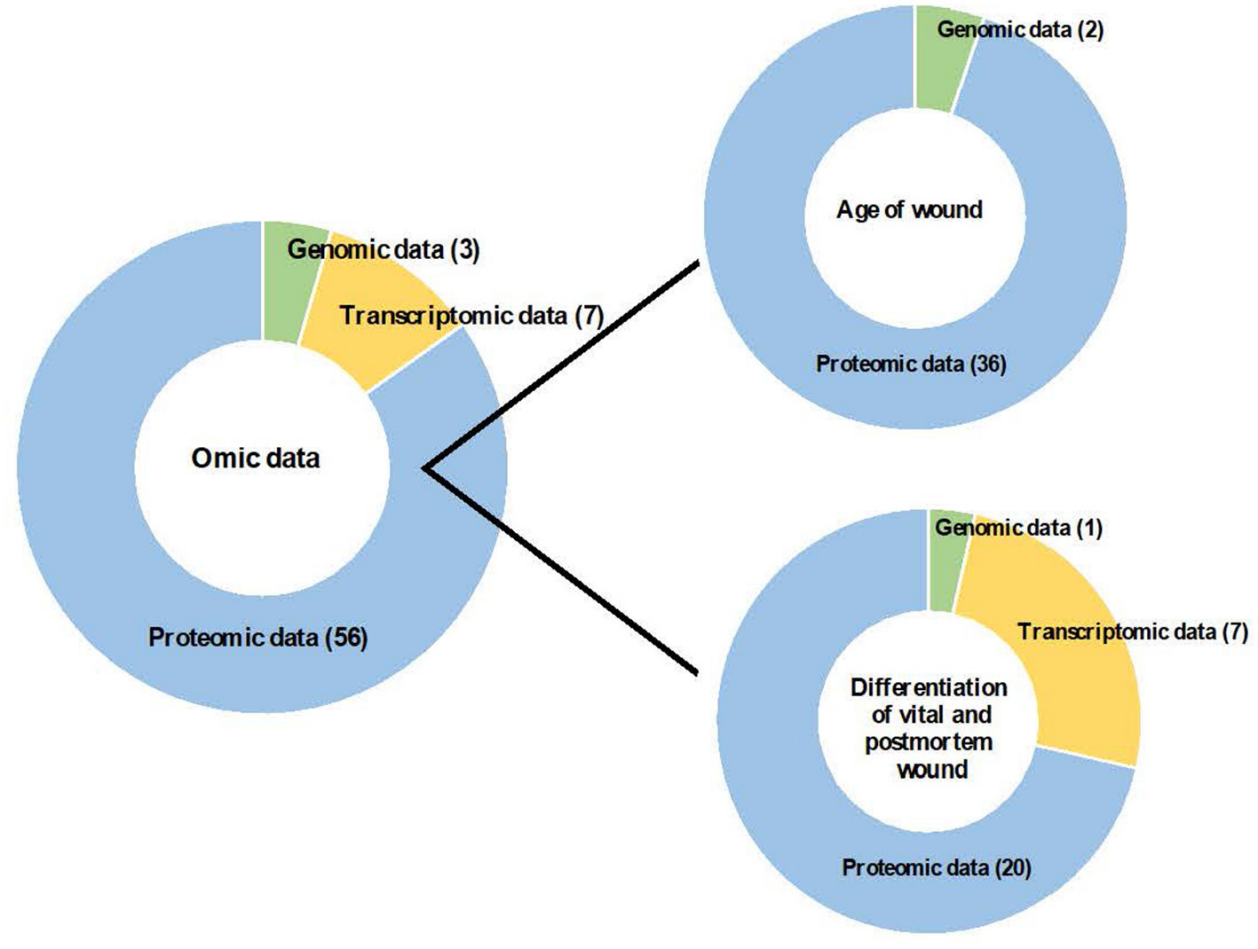
Contribution of omic sciences to the study of age of wound and differentiation of vital and post-mortem wound.

Finally, this search strategy identified 66 descriptive studies according to the following classification: (i) age of wounds (*n* = 38): genomic data (*n* = 2) and proteomic data (*n* = 36); and (ii) differentiation of vital and post-mortem wounds (*n* = 28): genomic data (*n* = 1), transcriptomic data (*n* = 7) and proteomic data (*n* = 20); that were included in this systematic review (Figure 2).

### Risk of Bias Assessment

According to the CASP risk of bias assessment, most studies (57.58%) were judged as “good” due to the considered variables, while 42.42% were judged as “poor” or “moderate,” primarily due to confounding variables not being considered (Tables 1, 2). Participants were recruited from few geographic regions, making it difficult to generalize beyond these regions. Overall, the quality of the literature was good.

**Table 1:** Risk of bias assessment: age of wounds^a^.

**Omics studies**	**Address a clearly focused issue**	**Acceptable cohort recruitment**	**Exposure accurately measured**	**Outcome accurately measured**	**Important confounding factors identified**	**Important confounding factors accounted for**	**Precise results**	**Believable results**	**Results fit with other available data**	**Overall quality score**
**Genomic data**										
Suárez-Peñaranda et al. (28)	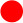	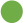	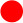	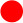	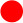	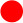	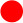	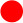	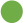	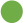
Betz et al. (29)	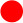	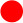	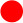	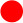	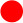	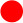	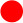	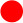	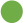	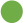
**Proteomic data**										
Hausmann et al. (30)	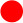	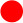	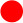	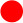	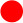	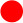	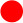	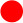		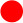
Tarran et al. (31)	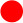	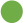	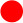	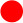	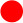	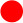	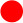	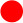	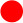	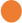
Betz et al. (32)	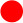	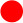	–	–	–	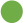	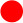	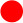	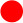	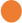
Betz et al. (33)	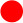	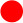	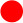	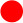	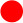	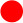	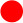	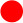	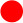	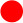
Balazîc et al. (34)	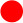	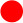	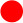	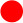	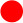	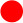	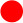	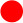	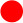	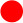
Van de Goot et al. (35)	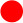	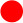	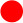		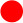	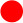	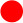	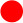	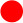	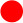
Guler et al. (36)	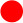	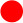	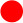	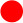	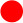	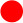	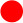	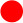	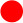	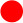
Kondo et al. (37)	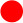	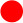	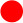	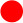	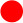	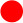	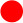	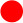	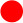	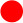
Fieghut et al. (38)	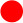	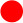	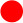	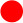	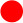	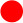	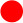	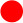	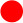	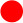
Dreßler et al. (39)	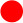	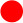	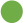	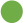	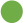	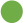	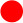	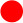	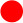	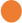
Dreßler et al. (40)	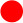	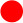	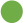	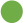	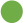	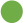	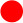	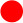	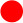	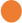
Dreßler et al. (41)	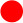	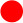	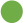	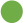	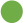	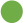	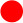	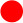	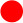	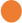
Dreßler et al. (3)	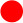	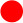	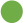	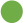	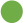	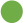	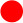	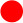	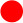	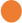
Dreßler et al. (42)	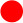	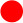	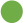	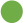	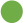	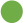	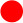	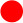	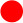	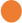
Betz et al. (43)	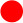	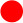	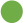	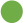	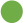	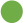	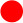	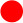	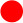	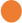
Fronczek et al. (44)	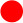	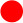	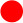	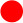	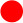	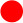	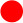	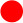	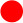	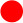
Grellner et al. (45)	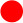	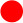	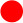	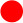	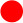	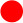	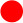	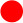	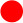	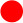
Grellner et al. (46)	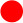	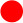	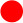	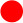	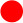	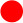	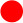	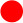	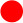	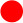
Birincioglu et al. (47)	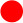	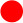	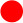	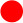	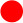	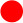	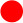	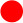	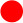	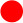
Takamiya et al. (48)	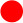	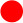	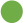	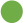	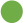	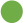	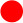	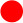	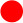	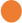
Kondo et al. (49)	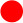	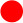	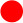	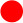	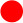	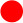	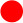	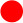	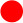	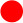
Ishida et al. (50)	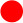	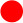	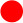	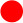	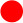	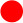	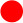	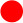	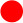	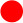
Ishida et al. (51)	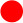	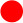	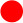	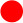	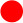	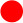	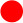	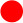	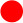	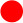
Kuninaka et al. (52)	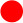	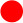	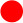	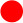	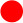	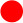	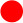	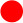	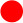	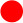
Jebur et al. (53)	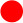	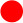	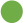	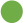	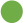	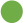	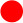	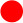	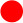	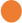
Bonelli et al. (54)	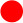	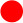	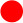	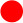	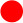	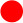	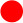	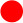	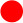	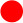
Kondo et al. (55)	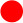	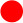	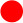	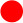	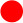	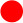	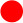	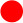	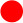	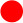
Grellner et al. (4)	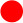	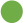	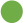	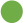	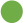	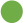	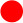	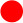	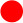	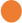
Yagi et al. (56)	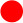	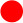	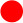	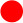	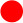	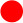	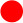	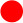	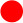	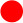
Hayashi et al. (57)	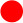	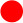	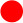	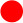	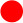	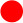	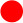	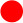	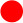	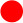
Ishida et al. (58)	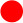	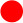	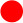	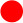	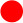	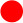	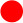	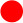	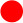	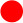
Ishida et al. (59)	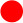	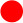	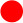	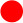	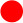	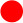	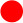	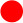	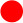	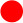
Ishida et al. (60)	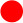	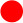	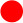	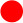	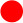	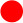	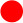	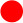	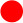	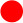
Ishida et al. (61)	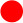	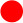	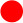	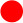	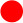	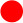	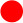	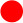	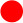	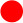
Dachun and Jiazhen (62)	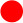	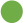	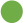	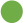	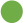	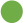	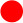	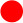	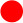	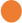
Betz (63)	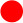	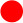	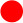	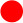	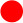	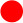	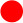	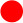	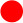	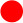

a *Data based on CASP-based risk of bias assessment*.

*Green, good risk of bias; Orange, moderate risk of bias; Red, low risk of bias*.

**Table 2:** Risk of bias assessment: differentiation of vital and post-mortem wounds^a^.

**Study**	**Address a clearly focused issue**	**Aceptable cohort recruitment**	**Exposure accurately measured**	**Outcome accurately measured**	**Important confounding factors identified**	**Important confounding factors accounted for**	**Precise results**	**Believable results**	**Results fit with other available data**	**Overal quality score**
**Genomic data**										
Grellner and Benecke (64)	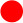	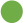	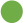	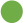	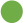	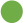	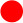	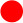	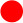	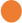
**Transcriptomic data**										
Xu et al. (65)	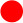	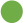	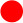	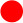	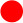	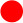	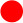	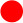	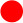	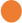
Ye et al. (66)	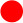	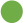	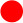	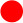	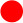	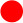	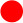	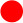	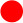	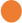
Qu et al. (67)	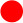	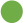	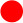	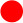	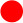	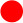	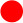	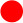	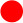	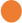
He et al. (68)	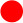	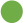	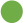	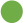	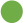	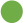	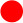	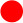	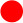	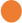
Liapi et al. (69)	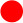	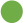	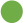	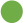	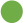	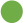	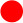	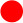	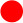	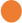
Neri et al. (70)	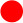	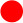	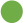	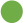	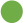	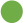	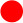	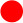	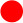	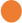
Kubo et al. (71)	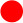	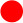	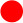	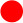	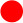	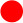	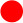	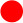	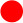	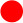
**Proteomic data**										
Ishida et al. (72)	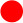	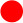	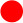	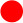	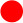	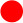	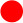	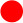	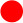	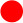
Prangenberg et al. (73)	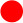	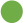	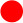	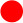	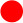	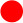	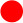	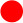	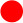	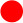
Bonelli et al. (74)	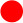	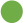	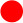	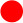	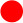	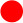	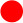	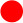	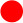	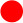
Oehmichen et al. (75)	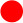	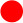	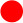	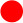	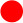	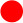	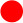	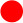	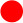	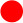
Turillazzi et al. (76)	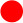	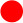	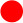	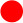	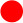	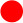	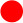	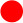	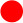	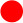
Gauchotte et al. (77)	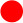	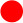	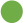	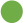	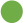	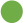	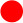	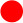	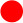	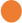
Legaz et al. (78)		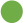	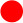	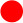	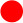	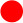	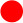	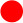	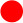	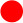
Hernández-Cueto et al. (79)	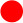	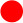	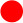	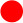	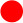	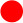	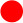	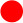	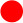	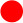
Montisci et al. (80)	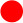	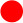	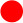	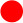	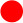	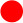	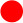	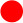	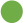	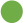
Ortiz-Rey et al. (81)	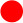	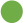	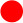	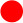	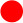	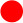	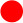	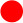	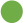	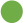
Ortiz-Rey et al. (82)	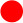	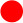	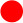	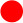	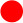	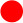	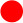	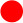	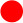	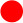
Legaz Pérez et al. (83)	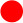	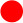	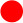	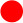	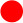	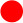	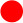	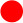	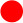	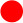
Yu-Chuan et al. (84)	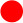	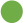	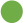	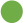	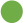	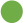	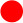	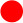	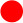	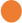
Ali (85)	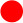	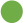	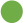	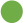	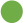	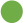	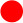	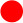	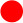	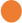
He and Zhu (86)	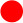	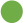	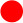	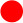	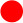	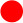	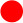	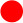	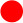	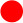
Bacci et al. (87)	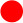	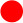	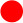	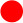	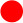	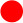	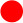	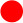	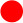	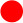
Peyron et al. (88)	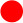	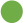	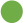	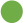	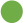	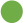	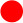	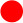	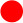	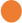
Kimura et al. (89)	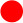	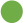	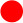	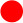	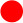	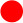	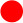	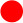	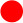	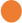
Bacci et al. (90)	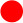	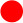	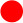	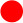	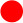	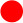	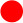	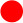	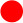	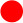
Focardi et al. (91)	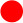	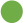	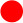	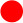	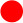	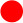	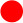	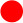	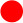	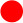

a *Data based on CASP-based risk of bias assessment*.

*Green, good risk of bias; Orange, moderate risk of bias; Red, low risk of bias*.

### Laboratory Methods

**Figure 3:**
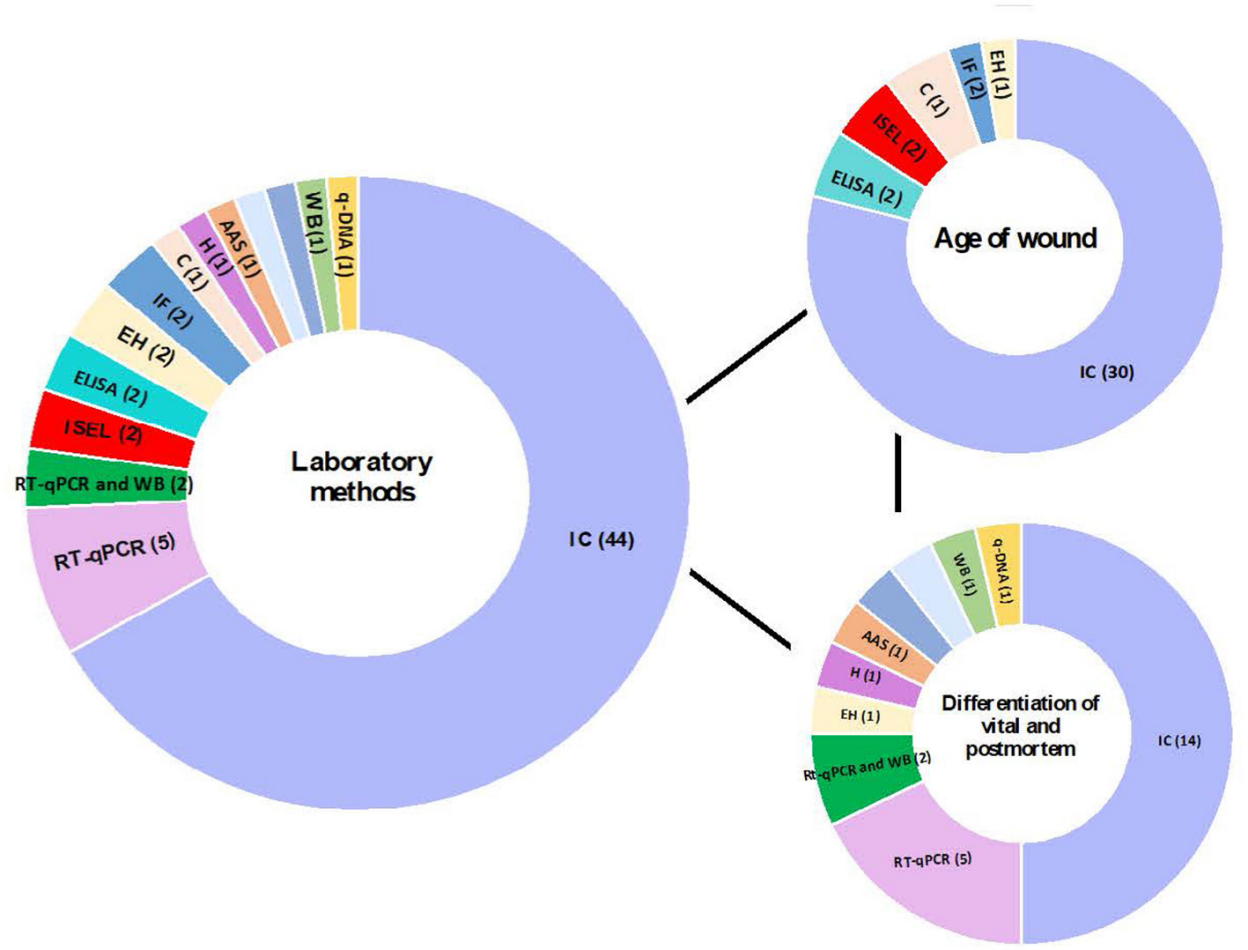
Contribution of different laboratory techniques to the analysis of age of wound and differentiation of vital and post-mortem wound. q-DNA analysis, quantitative DNA analysis; IC analysis, Immunohistochemical Analysis; EH analysis, Enzyme histochemical analysis; WB, western blot; AAS, Atomic absorption spectrometry; H analysis, Histological analysis; HPLC, liquid chromatography; MSI, Multiplex sandwich immunoassay.

The methods used varied between studies (Tables 3, 4; Figure 3). To determine the age of the wound, most studies (30/38) used inmunohistochemical analysis to detect different biomarkers involved in the healing wound. Two studies (28, 29) used *In Situ* Labeling of DNA fragments (ISEL) to detect DNA fragments. The other two studies used ELISA (45, 47), and the other two used immunofluorescence analysis (51, 52). Finally, a study (54) used Cytochemistry analysis, and another study (63) used enzyme histochemical analysis (Table 3). On the other hand, most studies (14/28) used inmunohistochemical analysis to differentiate between vital and post-mortem wounds. Five studies (65, 66, 69–71) used RT-qPCR and two studies (67, 68) used RT-qPCR and western blot to detect transcriptomic data. Other seven studies used different techniques: q-DNA analysis (64), enzyme histochemical analysis (79), histological analysis (85), atomic absorption spectrometry (84), liquid chromatography (86), multiplex sandwich immunoassay (88) and western blot (89) (Table 4).

**Table 3:** Age of wound in post-mortem studies^a^.

**Study**	***N***	**Age (years)**		**PI (hours)**		**Male/Female**	**Population analyzed**	**Type of wound**	**Biomarker analyzed**	**Analyses technique**
		**Range**	**Mean**	**Range**	**Mean**					
**Genomic data**										
Suárez-Peñaranda et al. (28)	30	28–76	46.7	n.i.	n.i.	18/12	Spain	Wounds inflicted with a scalpe.	Apoptotic keratinocytes	ISEL
Betz et al. (29)	56	17–75	52	<72	n.i.	n.i.	Germany	Lacerations, stab wounds and surgical wounds.	Apoptotic fibroblastic cell	ISEL
**Proteomic data**										
Hausmann et al. (30)	82	17–75	52	24–96	n.i.	n.i.	Germany	Lacerations, stab wounds and surgical wounds.	p53	IC
Tarran et al. (31)	13	2–70	38	n.i.	n.i.	6/5	Australia	Thermal burns	p53	IC
Betz et al. (32)	53	15–92	54	<72	n.i.	n.i.	Germany	Surgical wounds, lacerations, stab wounds, heamtomas and abrasions.	Fibronectin	IC
Betz et al. (33)	56	15–92	54	<72	n.i.	n.i.	Germany	Surgical wounds, stab wounds and lacerations.	Tenascin	IC
Balazîc et al. (34)	48	n.i.	n.i.	n.i.	n.i.	n.i.	Slovenia	Gunshot wounds	Fibronectin	IC
Van de Goot et al. (35)	322	0–95	n.i.	0–48	n.i.	n.i.	Netherlands	Only skin wound samples that are the result of “blunt force trauma”.	Fibronectin, CD62p and Factor VIII	IC
Guler et al. (36)	170	15–85	39.44	4–24	18	74/15	Turkey	Gunshot wounds, blunt injuries, Sharp weapon injuries, and surgical excisions.	Tenascin and ubiquitin	IC
Kondo et al. (37)	55	8–75	40.6	<72	n.i.	n.i.	Germany	Stab wounds, incised wounds, surgical wounds and lacerations.	Ubiquitin	IC
Fieguth et al. (38)	38	12–89	44	24–48	n.i.	21/17	Germany	Neck soft tissue	Myoglobin, Fibronectin, C5b-9, MRP14	IC
Dreßler et al. (39)	132	n.i.	n.i.	n.i.	n.i.	n.i.	Germany	Injured skin.	ICAM-1 (CD54)	IC
Dreßler et al. (40)	65	n.i.	n.i.	n.i.	n.i.	42/23	Germany	Lacerated wounds, incised wounds and excoriations.	ICAM-1 (CD54)	IC
Dreßler et al. (41)	97	n.i.	49.5	n.i.	n.i.	n.i.	Germany	Lacerated/contused wounds, incised wounds, and excoriations.	Selectins	IC
Dreßler et al. (3)	97	n.i.	n.i.	n.i.	n.i.	n.i.	Germany	Injured skin	VCAM-I	IC
Dreßler et al. (42)	194	n.i.	n.i.	n.i.	n.i.	n.i.	Germany	Injured skin	VCAM-I, ICAM-1, P-selectin, E-selectin and L-selectin	IC
Betz et al. (43)	74	n.i.	n.i.	n.i.	n.i.	n.i.	Germany	Surgical wounds, lacerations, and stab wounds after surgical treatment.	Collagen types I and VI	IC
Fronczek et al. (44)	101	17–80	37	n.i.	n.i.	52/49	Netherlands	Bruises, abrasions, bites, stabs, scratches, and firework.	Collagen III, collagen IV and α-smooth muscle actin.	IC
Grellner et al. (45)	48	n.i.	45.8	n.i.	n.i.	33/15	Germany	Stab and incised wounds.	IL-1β, IL-6, TNF- α	ELISA
Grellner et al. (46)	105	3–93	51.1	4–192	56.8	n.i.	Germany	Skin wounds are caused by sharp force.	IL-1β, IL-6, TNF- α	IC
Birincioglu et al. (47)	50	10–80	41.12	n.i.	n.i.	44/6	Turkey	Firearms, penetrating trauma by sharp objects and blunt trauma.	IL-1β, IL-6, TNF-α and EGF.	ELISA
Takamiya et al. (48)	121	n.i.	n.i.	n.i.	n.i.	58/63	Japan	Incised wounds, stab wounds, laceration and contusions.	IL-2, IL-4, IL-6, IL-8, IL-10, GM-CSF, IFN-γ and TNF- α	IC
Kondo et al. (49)	50	7–77	48.8	<72	n.i.	n.i.	Germany	Skin wounds.	IL-8, MCP-1, and MIP-1α	IC
Ishida et al. (50)	53	8–75	40.6	<72	n.i.	n.i.	Germany	Stab wounds, incised wounds, surgical wounds and lacerations.	CD45 and collagen type 1	IC
Ishida et al. (51)	52	8–75	40.6	<72	n.i.	n.i.	Germany	Stab wounds, incised wounds, surgical wounds and lacerations.	CD34/Flk-1	IF
Kuninaka et al. (52)	53	8–75	40.6	0–72	n.i.	n.i.	Germany	Stab wounds, incised wounds, surgical wounds or lacerations.	CD11c and HLA-DRα	IF
Jebur et al. (53)	88	n.i.	n.i.	n.i.	n.i.	n.i.	Irak	Lacerated skin wound.	Tryptase, IL-1 and IL-6	IC
Bonelli et al. (54)	75	10–97	n.i.	n.i.	n.i.	55/20	Italy	Surgical wounds, lacerations and abrasions.	Tryptase and cymase	C
Kondo et al. (55)	40	8–75	40.6	<72	n.i.	28/12	Germany	Stab wounds, incised wounds, surgical wounds and lacerations.	IL-1α	IC
Grellner et al. (4)	n.i.	n.i.	n.i.	n.i.	n.i.	n.i.	Germany	Skin wounds.	TGF-α, and TGF-β	IC
Yagi et al. (56)	44	0–86	56.2	<72	n.i.	n.i.	Japan	n.i.	CD14	IC
Hayashi et al. (57)	53	8–75	40.6	<72	n.i.	n.i.	Germany	Stab wounds, incised wounds, surgical wounds and lacerations.	VEGF	IC
Ishida et al. (58)	58	8–75	48.6	<72	n.i.	n.i.	Germany	Stab wounds, incised wounds, surgical wounds and lacerations.	ORP150	IC
Ishida et al. (59)	55	7–83	45.8	<72	n.i.	n.i.	Germany	Stab, incised, surgical or laceration wounds	MMP-2 and MMP-9	IC
Ishida et al. (60)	60	7–83	46.5	<72	n.i.	n.i.	Germany	Stab wounds, incised wounds, surgical wounds and lacerations.	Cyclooxygenase-2	IC
Ishida et al. (61)	55	7–83	45.8	<72	n.i.	n.i.	Germany	Stab wounds, incised wounds, surgical wounds or lacerations.	Aquaporin-1 and aquaporin-3	IC
Dachun and Jiazhen (62)	8	n.i.	n.i.	n.i.	n.i.	n.i.	China	Gunshot wounds, lacerations and incisions.	Non-specific esterase	IC
Betz (63)	221	15–94	50	<96	n.i.	148/73	Germany	Lacerations, surgical or stab/cut wounds.	Nonspecific esterases, Acid phosphatase, ATPase, Aminopeptidase and Alkaline phosphatase.	EH

a *n, number of individuals or samples*.

*PI, post-mortem interval; n.i., not indicated; ISEL, In situ end-labeling of DNA fragments; IC, Immunohistochemical Analysis; IF analysis, Immunofluorescence analysis; C, Cytochemistry analysis; EH, Enzyme histochemical analysis; ELISA, Enzyme-Linked Immunosorbent Assay*.

**Table 4:** Differentiation of vital and post-mortem wound in post-mortem studies^a^.

**Omic studies**	***N***	**Age (years)**		**PI (hours)**		**Male/Female**	**Population analyzed**	**Type of wound**	**Biomarker analyzed**	**Analyses technique**
		**Range**	**Mean**	**Range**	**Mean**					
**Genomic data**										
Grellner and Benecke (64)	24	n.i.	47.8	n.i.	n.i.	20/4	Germany	Strangulation marks	DNA	q-DNA
**Transcriptomic data**										
Xu et al. (65)	6	26–52	37.7	28–46	35.5	3/3	China	Traumatic injuries of traffic accident	Cxcl1, Jun, Fos, IL-6, and Sfrp2.	RT-qPCR
Ye et al. (66)	21	18–56	31.2	31–72	52.4	11/10	China	Traumatic injuries of traffic accident.	IL-6 and IL-20	RT-qPCR
Qu et al. (67)	6	27–60	41.5	39–96	64.2	3/3	China	Traumatic injuries	ATF3 and BTG2	RT-qPCR and WB.
He et al. (68)	5	n.i.	n.i.	n.i.	n.i.	n.i.	China	Traumatic injuries of traffic accident	CXCL1 and CXCR2	RT-qPCR and WB.
Liapi et al. (69)	19	n.i.	n.i.	n.i.	n.i.	n.i.	Germany	Stab wounds.	GAPDH, PGK1, YWHAZ and PPIA.	RT-qPCR
Neri et al. (70)	64	n.i.	n.i.	12–24	n.i.	n.i.	Italy	Ligature marks of suicidal hanging.	miRNA 92a-3p, 125a-5p, 214-3p, 125b-5p, 103a-3p	RT-qPCR
Kubo et al. (71)	48	20–88	63.1	<72	n.i.	26/22	Japan	Burned skins, abrasion and bruise skins.	Aquaporin-3	RT-qPCR
**Proteomic data**										
Ishida et al. (72)	56	0–89	56.1	8–72	30.5	33/23	Japan	Ligature marks of suicidal hanging and strangulation.	Aquaporin-1 and aquaporin-3	IC
Prangenberg et al. (73)	30	19–95	54.6	n.i.	n.i.	19/11	Germany	Dried skin abrasions, frost erythema, laceration, stab wound, gunshot wound, and strangulation mark.	Aquaporin-1 and aquaporin-3	IC
Bonelli et al. (74)	20	22–79	48.3	n.i.	n.i.	19/1	Italy	Surgical wounds, lacerations and abrasions.	Tryptase and cymase	IC
Oehmichen et al. (75)	64	6–71	36.75	<48	n.i.	35/29	Germany	n.i.	Tryptase and esterase NAS-DClAE	IC
Turillazzi et al. (76)	70	20–50	29.16	n.i.	n.i.	40/30	Italy and Spain	Ligature marks of suicidal hanging.	Tryptase, CD15 and IL-15.	IC
Gauchotte et al. (77)	92	n.i.	n.i.	n.i.	n.i.	n.i.	France	Stab.	FVIII, CD15 and tryptase	IC
Legaz et al. (78)	15	21–47	33.6	19–36	n.i.	12/3	Spain	Ligature mark of suicidal hanging.	Fibronectin, cathepsin-D, and P-selectine.	IC
Hernández-Cueto et al. (79)	53	14–82	36.7	n.i.	n.i.	46/7	Germany	Incision wounds.	Cathepsin D	EH
Montisci et al. (80)	40	36–80	58.48	n.i.	n.i.	25/15	Italy	Skin fragmens.	Cathepsin-D	IC
Ortiz-Rey et al. (81)	24	35–76	57.1	n.i.	n.i.	13/11	Spain	Surgical incisions.	P-selectin	IC
Ortiz-Rey et al. (82)	48	33–76	59.2	2–16	7.7	27/21	Spain	Incised wounds	Fibronectin and tenascin	IC
Legaz Pérez et al. (83)	71	12–82	35.7	19–36	n.i.	61/10	Spain	Ligature mark of suicidal hanging	Fe, Zn, Mg, Ca, P-selectin, and cathepsin D.	IC
Yu-Chuan et al. (84)	14	20–45	n.i.	24–72	n.i.	12/2	China	Lacerations, contusions and stab wounds.	Fe, Zn, Mg, Cu, K, and Na	AAS
Ali (85)	19	n.i.	n.i.	n.i.	n.i.	n.i.	UK	Ligature marks, electric marks, edges of stab wounds, burns, and bruises.	Collagen	H
He and Zhu (86)	7	10–65	29	4–24	13,29	5/2	China	Lacerations and skin incisions.	LTB_4_	HPLC
Bacci et al. (87)	40	19–79	n.i.	n.i.	n.i.	30/10	Italy	Skin lesions	TNF-α	IC
Peyron et al. (88)	24	n.i.	n.i.	n.i.	n.i.	n.i.	France	Lacerations, stab wounds and gunshot wounds.	IFN-γ, IL-1β, IL-2, IL-4, IL-6, IL-8, IL-10, IL-12p70, IL-13,TNF-α.	MSI
Kimura et al. (89)	4	16–61	39.5	≤ 24	24	3/1	Japan	Compression mark, stab wound, and ligature mark.	LC3-II and p62	WB
Bacci et al. (90)	80	3–86	50	24–48	n.i.	56/24	Italy	Car accident, fall, homicide, or hanging	MHC-II and CD1a	IC
Focardi et al. (91)	20	3–89	n.i.	24–48	41	14/6	Italy	Hanging mark wounds.	MHC-II and CD1a	IC

a *n, number of individuals or samples*.

*PI, post-mortem interval; n.i., not indicated; q-DNA analysis, quantitative DNA analysis; IC analysis, Immunohistochemical Analysis; EH analysis, Enzyme histochemical analysis; WB, western blot; AAS, Atomic absorption spectrometry; H analysis, Histological analysis; HPLC, liquid chromatography; MSI, Multiplex sandwich immunoassay*.

## Wound Characterization in Post-Mortem Forensic Studies

### Age of Wounds

When faced with a wound, one of the main tasks of the forensic pathologist is to determine how long the victim survived after the wound was inflicted (92). Healing of a skin wound begins immediately after injury and consists of three phases: inflammation, proliferation, and maturation, which involve interactions between various types of cells and soluble factors (20, 93–96).

**Figure 4:**
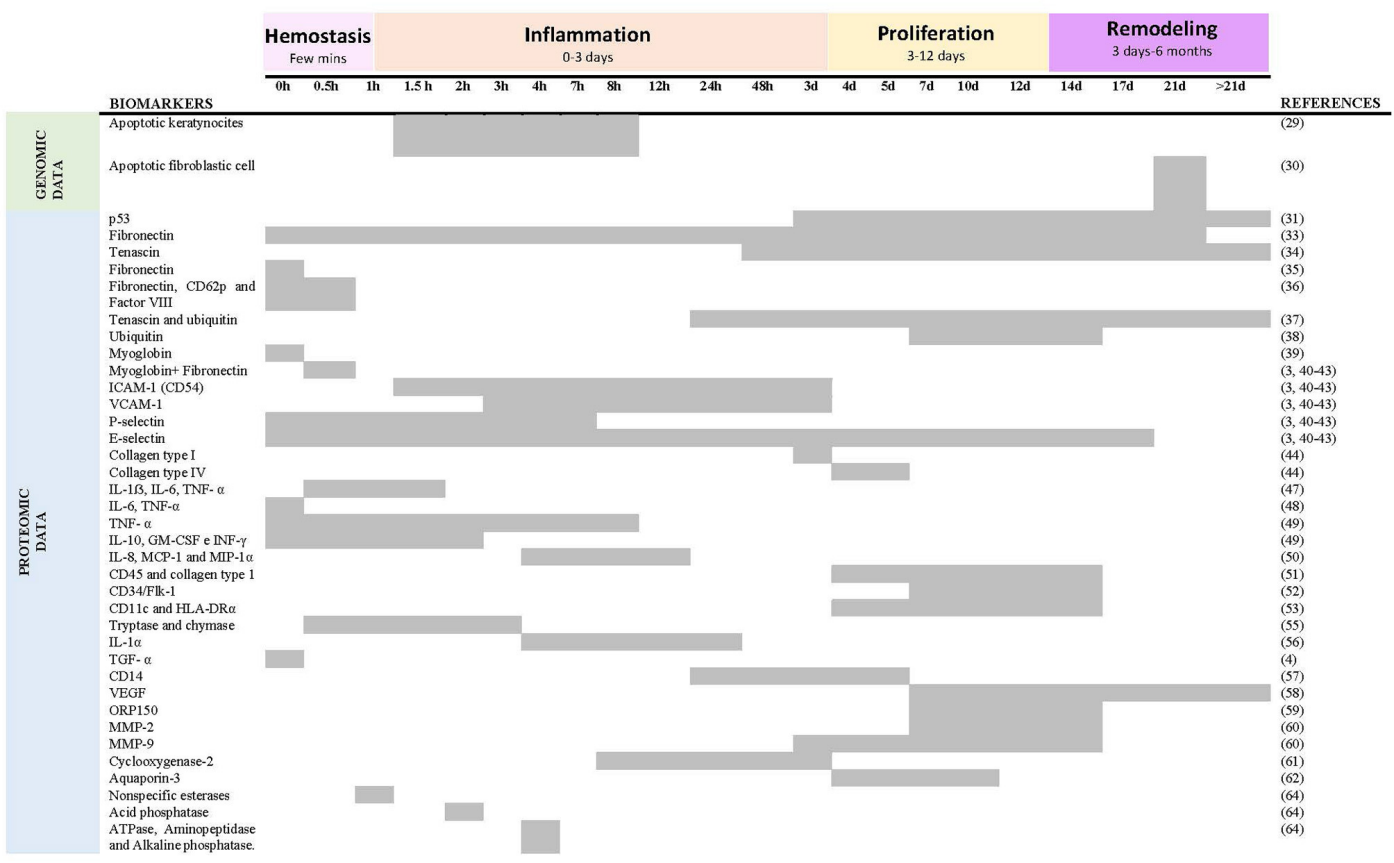
Summary of reactivity of age biomarkers respect to time after skin injury. Five studies (31, 44, 45, 53, 62) were excluded because they do not place the appearance and/or quantification of a marker on a timeline about the age of the wound.

A total of 38 descriptive studies on the determination of the age of vital wounds have been reviewed (Table 3). The studies that place the appearance and/or quantification of specific markers on a timeline referring to the age of the wound in comparison with the group used as control have been collected in Figure 4.

Two studies analyze genomic data using *in situ*-end labeling (ISEL). They detect and quantify nuclear DNA fragments that appear as a consequence of direct cell injury or during programmed cell death (apoptosis), thus providing information on the wound healing process and, as a consequence, on its age. Suárez-Peñaranda et al. (28) limited their research to incisional lesions, showing that the finding of apoptosis in keratinocytes of the dermis can be an early vitality marker. However, they did not find variations in the level of positive cells when the age of the wound increases. Therefore, the validity of this marker to determine the age of the wound is doubtful. On the contrary, another study (29) analyzed lacerations, stab wounds, and surgical wounds, concluding that a rapid increase in the number of fibroblasts occurs during wound healing after ~3 weeks.

Thirty-six studies associate wound age estimation with the detection and quantification of different proteins (proteomics data) mainly involved in the inflammation phase of the wound healing process. Two studies analyzed the expression of the protein p53, a protein responsible for stopping the cell cycle when it detects DNA damage, thus allowing the repair of damaged DNA or inducing apoptosis.

Hausmann et al. (30) analyzed lacerations, stab wounds, and surgical wounds and stated that it could be expected to find a significant number of fibroblasts positive to p53 (*r* ≥ 0.2) in wounds of at least 3 days of age, while when the values of are >0.5, the post-infliction interval is at least 8 days. On the other hand, Tarran et al. (31) explored the expression of p53 in antemortem and post-mortem material, concluding that more studies are necessary to determine the minimum survival period necessary for a burn wound to express the protein mentioned above and to determine if its expression can occur in uninjured skin such as a result of agonizing stress.

Two studies of Betz et al. (32, 33) analyze two glycoproteins of the extracellular matrix, fibronectin, and tenascin, respectively, in surgical wounds, stab wounds, lacerations, bruises, and abrasions. These glycoproteins support the adhesion of different cells such as fibroblasts and endothelial cells, participating in the early stages of the wound healing process. The immunohistochemical investigations of these authors demonstrated that fibronectin allows differentiation between wounds of less than two and more than 3 weeks, while tenascin appears for the first time 2 days after the injury, around fibroblastic cells, observing a decrease in the intensity of tenascin staining with increasing age, tenascin still being present in wounds up to 1.5 months. Similarly, Balažic et al. (34) also studied fibronectin's expression, but unlike the previous ones, they analyzed gunshot wounds. Their results indicate that fibronectin is a reliable marker of the vitality and age of wounds with a short survival time (few minutes).

van de Goot et al. (35) analyzed the fibronectin glycoprotein together with CD62p and coagulation factor VIII. Fibronectin is responsible for forming a clot at the site of the injury, promoting the spread of platelets, as well as the migration of neutrophils, monocytes, fibroblasts, and cells endothelial cells, CD62p and factor VIII being present on the surface of the latter a few minutes after a wound occurs. They observed a significant increase of the three markers in wounds of 15–30 min compared to the uninjured control samples. Another study (36) studies the expression of tenascin together with that of ubiquitin, a cytokine-like protein with anti-inflammatory properties expressed in neutrophils, leukocytes, macrophages, and fibroblasts in the wound area. They analyzed gunshot wounds, blunt injury, and sharp injury, showing no relationship between the type of wound and the determination of age by tenascin and ubiquitin. They found a positive correlation between the number of positive cells for both markers and the age of the wound. Tenascin was positive in 91.8% of the cases with a wound age > 24 h and negative in 98.3% of the cases with a wound age <24 h. In contrast, ubiquitin was positive in 4.25% of the cases with a wound age <24 h and in 26.14% of the cases with a wound age > 24 h. When the wound age was > 40 days, the fibroblasts still expressed ubiquitin, but not tenascin.

Kondo et al. (37) found significant differences in the expression of ubiquitin between different age groups of the wound, the wounds between 7 and 14 days old showed the highest expression of ubiquitin, decreasing this from day 17. Other authors (38) examined lesions in the neck, indicating that an accumulation of myoglobin indicates a lesion with a survival time of a few minutes. In contrast, if fibronectin is also detected in the same lesion, the post-infliction interval is several minutes. Positive C5b-9 reactions indicate that death did not occur during strangulation but occurred afterward.

Different investigations of Dreßler et al. (3, 39–42) analyzed the expression of different adhesion molecules: ICAM-1, VCAM-1 and selectins. VCAM-1, ICAM-1, P-selectin, and E-selectin are endothelial adhesion molecules whose expression requires activation by lipopolysaccharides and cytokines, especially IL-1β and TNF-α, which are released in the wound healing process. Therefore, these molecules are essential in the inflammation phase of the wound. Immunohistochemical investigation did not reveal strong expression of ICAM-1 by endothelial cells and keratinocytes until approximately a minimum of 1.5 h after injury and up to a maximum of 3.5 days, the intensity of VCAM-1 increased with the increasing number of blood vessels, observing a strong intensity 3 h after the infliction of the wound and up to 3.5 days later. On the other hand, they also determined that L-selectin is not valid for estimating age, while P-selectin was found in an interval between 3 min and 7 h after injury, and E-selectin was between 1 h and 17 days.

Two more studies look at collagen to estimate the age of the wound. On the one hand, Betz et al. (43) state that network structures that react positively for type I or VI collagen indicate a wound age of at least 5–6 days and 3 days, respectively. On the contrary, Fronczek et al. (44) studied the presence of type I collagen in blood vessels for wound estimation, instead of network structures, in addition to the presence of type III, IV, and Alpha-smooth muscle actin collagen.

Two studies (45, 46) analyzed IL-1β, IL-6, and TNF-α by ELISA and immunohistochemical techniques, respectively, obtaining more precise results with immunohistochemical techniques. The three proinflammatory cytokines proved to be useful markers for determining a lesion onset interval of up to a few hours. The first increase in reactivity could be noted for IL-1β, IL-6, and TNF-α, almost simultaneously after 15 ± 20 min, their expression changing after ~1 ± 1.5 h.

A more recent study (47) indicates IL-6 and TNF-α as early phase markers indicating a wound age of <30 min. However, they consider that the usefulness of IL-1β and EGF should be reevaluated.

In correlation with the previous studies, another study (48) showed a significant increase in TNF-α in survival times of <30 min. IL-8, the most abundant cytokine in this study, has been shown to originate from keratinocytes, fibroblasts, endothelial cells, and neutrophils. In addition, IL 8 proliferates keratinocytes and acts as a potent chemokine for neutrophils and lymphocytes. IL-2 promotes T lymphocyte proliferation and interacts with IFN-γ in the production of IL-8 mRNA in keratinocytes. On the other hand, IL-4 is believed to proliferate fibroblasts. Significant expressions of IL-6, IL-8, IFN-γ, and TNF-α have significant effects on dermal wound healing.

A sample of 50 wounds of different ages from autopsies observed polymorphonuclear cells with positive reactions to IL-8 and the inflammatory proteins MCP-1 and MIP-1alpha in wounds of 4–12 h (49).

Fibrocytes are mesenchymal progenitors that co-express cell antigens and fibroblast products such as CD45 and type I collagen and are involved in tissue repair after injury. Ishida et al. (50) consider that the determination of fibrocytes from the CD45 and type I collagen markers have great precision and objectivity when estimating the age of a wound. They demonstrated the appearance of fibrocytes in human skin wounds with a wound age of at least 4 days, suggesting a number of fibrocytes > 15 and a wound age of 9–14 days. After demonstrating the usefulness of fibrocytes in determining wound age, a study by the same authors (51) investigated the utility of endothelial precursor cells (EPC) that contribute to vasculogenesis, a process essential for the survival of growing, injured and ischemic tissue. Thus, after analyzing 52 skin wounds from autopsies using immunofluorescence analysis, they determined that the number of CPE was significantly high in wounds between 7 and 14 days with more than 20 CPE in most cases, while it decreased below 15 CPE in wounds of more than 14 days. Third, these researchers (52) hypothesized that dendritic cells (DCs) were closely related to the onset of the immune response after injury, and again by immunofluorescence, they determined that a count > 50 DC in a wound sample would indicate an age of same between 4 and 14 days.

A more recent study (53) studied tryptase as a marker of mast cell activation as a factor of wound vitality and two proinflammatory cytokines (IL-1 and IL-6 to determine the age of the wound. Using immunohistochemical techniques, they found a positive correlation between infiltration with mast cells (mast cells tryptase) and the passage of time.

A total of 75 vital skin lesions were examined by Bonelli et al. (54). Their investigations demonstrated a progressive and significant increase in the number of neutrophils with the time elapsed between injury and death. Mast cell density progressively increased to a maximum in vital injuries between 1 and 3 h before death, decreasing after that.

On the other hand, a group of authors (55) stated that in six out of ten wounds aged between 4 h and 1 day, the proportions of infiltrating cells positive for IL-1α were higher than 30%, being lower than the said percentage in wounds between 1.5 and 21 days.

The stimulation and regulation of angiogenesis are among the most important effects of TGF-α in the wound healing process. At the same time, TGF-β1 has chemotactic activity and promotes extracellular matrix synthesis (collagen, fibronectin, tenascin), present in the three phases of healing. Grellner et al. (4) observed an increase in TGF-α reactivity from a wound age of 10–20 min, especially in the middle epidermal areas of the spinous layer. Otherwise, TGF-β1 was detected in all phases of wound repair, except for the lesions with the shortest survival time.

Another study (56) indicated that CD14 might be a helpful marker for estimating wound age between 1 and 5 days in forensic practice.

In cutaneous wound healing, the vascular endothelial growth factor (VEGF) plays a vital role as it is a crucial angiogenic factor for forming new granulation tissue in the proliferative phase. Some authors (57) suggest that a VEGF positive ratio > 50% indicates a wound age > 7 days.

Overexpression of the ORP150 gene by adenovirus vectors has accelerated wound healing by modulating VEGF (14). Ishida et al. (58) suggested that a positive ORP150 ratio > 50% indicates a wound age of 7–14 days.

In addition to growth factors, cytokines, and adhesion molecules, wound healing processes also involve matrix metalloproteases (MMPs). MMP-2 and MMP-9 bind gelatin, collagens, and laminin, with MMP-9 participating in the epithelialization process and early repair events, while MMP-2 plays a key role in the prolonged remodeling stage. A study (59) has shown that probably several MMP-2 positive macrophages > 20 indicate a wound age of between 7 and 12 days, while many MMP9- + cells > 30 would indicate a wound age between 3 and 14 days. Therefore, MMP-2 and MMP-9 would be useful markers of the proliferative phase of skin wound healing. On the other hand, IL-1 could positively regulate the gene expression of these two metalloproteases. One more study by Ishida et al. (60) suggested that a positive COX-2 ratio > 40% indicates a wound age of 8 h−3 days. The same authors (61) immunohistochemically examined the expression of AQP-1 and AQP-3 in human skin wounds by showing their participation in the migration of keratinocytes and endothelial cells, among others. Thus, they determined that a number of AQP-3 + cells > 300 possibly indicates a wound age of between 5 and 10 days.

Two studies analyze non-specific esterases for determining the age of the wound. On the one hand, some authors (62) quantified non-specific esterase (NSE) in injured skin using a microspectrophotometric scanning technique, indicating that it applied to medico-legal practice for determining the age of the wound. On the other hand, another study (63) showed that NSE activity increased ~1 h after injury, followed by changes in acid phosphatase at ~2 h, and changes in aminopeptidase, ATPase, and alkaline phosphatase activity at ~4 h, changes that did not they were evidenced in post-mortem wounds.

### Differentiation of Vital and Post-mortem Wounds

To assess the survival time from wound age estimation, the forensic pathologist must differentiate antemortem from post-mortem wounds. In this field, scientists investigate relevant markers of vital origin (22). Therefore, 28 articles have been selected that analyze different vitality markers to differentiate between injuries before and after death (Table 4).

Grellner and Benecke (64) used a genomic technique such as DNA quantification to analyze strangulation marks, concluding that quantitative changes in the DNA content of the grooves are not significant as a sign of vitality in strangulation.

On the other hand, seven studies analyzed transcriptomic data using the RT-qPCR technique. A study (65) discussed the possibility of using this technique to reveal differentially expressed genes (DEGs) as possible markers of vital reactions. They evaluated the results by studying five DEGs in wounds from human autopsies, observing how RNA expression levels of Cxcl1, Jun, Fos, and IL-6 increased in post-mortem human skin wounds compared to intact skin the Sfrp2 expression.

Another study (66) investigated IL-6 and IL-20 mRNA expression in mouse and human skin wounds. In animals, they found that the expression of IL-6 and IL-20 was more regulated in the contused area of the skin than in intact skin and post-mortem bruised skin. These results were validated by examining post-mortem human skin tissues, in which they were level. IL-6 and IL-20 mRNA were significantly higher in injured regions compared to intact ones.

Qu et al. (67) also analyzed the expression levels of ATF3 and BTG2 in human and mouse skin wounds. The protein levels examined by western blot showed no changes in the expression levels of both proteins between wounded and intact skins. However, the mRNA levels demonstrated a higher ATF3 and BTG2 in mouse skins with an antemortem contusion than intact skin and with post-mortem contusion. In human skin samples from forensic autopsies, increased levels of ATF3 mRNA were detected up to 48 h after the autopsy, but no differences were found between injured and intact skin for BTG2. ATF3 can be considered a potential marker for a vital skin contusion reaction, but BTG2 cannot.

Another study (68) analyzed mRNA levels in skin wounds in mice and humans, in this case, CXCL1 and CXCR2 proteins. As in the previous study, the western blot analysis of protein levels did not show differences between wounded and intact skin. The mRNA levels demonstrated higher CXCL1 and CXCR2 in bruised mouse and human skin compared to intact skin.

Liapi et al. (69) examined the effect of RNA integrity on reference gene expression stability for future normalization of relative qPCR data from intact skin and post-mortem wounds. Thus, GAPDH and PGK1 were classified as two reference genes stably expressed in post-mortem skin tissues, while YWHAZ and PPIA increased the variation in gene expression, so they should be excluded as reference genes.

Other authors (70) demonstrated an increase in the expression of different miRNAs recognized as regulators of the inflammatory response in skin lesions in wounded skin from people who died by hanging compared to healthy skin. Their data confirm that miRNA expression in traumatic skin wounds is related to an act of regulation of the inflammatory phase aimed at inhibiting intracellular signals activated by the production of inflammatory cytokines, even in cases of lesions that develop in a short time.

Another study (71) observed that, both in animal models and in cases of human autopsies, there was a significant difference in the expression of the AQP3 gene between pre and post-mortem burned skin. They, therefore, suggested that the expression of the dermal gene AQP3 was increased to maintain water homeostasis in response to dehydration from burns.

Twenty studies analyzed proteomic data to study vital markers in the differentiation of vital and post-mortem wounds. Ishida et al. (72) studied the expression of aquaporins AQP1 and AQP3 in suicide hanging and strangulation ligation marks using immunohistochemical techniques. The authors found no difference in AQP1 expression between compressed neck skin and uninjured skin. However, they observed that AQP3 was expressed in antemortem ligation mark keratinocytes obtained from forensic cases' autopsies compared to intact skin. Other authors (73) also analyzed AQP1 and AQP3 in strangulation marks and examined thermal injuries, gunshot wounds, and frostbite erythema. Like the previous ones, they did not find significant differences between injured and non-injured skin about the expression of AQP1. However, they did find a higher expression of AQP3 in epidermal keratinocytes in all types of lesions.

Bonelli et al. (74) determined that the mast cell density (positive tryptase and cymase) in vital lesions, observing that it was significantly higher in healthy controls and in post-mortem lesions.

Another study (75) analyzed skin wounds from 64 human cadavers to determine whether skin mast cells are activated during the very early phase of human wound healing. He compared the number of tryptase-reactive mast cells, which do not lose all their enzymatic activity during the degranulation process, with the number of naphthol AS-D chloroacetate esterase (NAS-DCIAE) positive mast cells, which lose their total enzymatic activity. In victims who survived the injury for <60 min, the average number of NAS-DCIAE-reactive mast cells along the wound margin was significantly lower than the number of tryptase-reactive mast cells. The findings of this study show that mast cells experience a very early loss of NAS-DCIAE activity at wound margins; thus, it appears to be an early cellular marker of wound survival.

On the other hand, Turillazzi et al. (76) investigated the immunohistochemical expression of a panel of cytokines and inflammatory cells in skin samples from an autopsy of hanging cases to evaluate whether the mark and signs of hanging occurred before or after the death of the victim. They conclude that tryptase and IL-15 can complement the determination of the vitality of CD15-based ligation marks with the precision necessary for forensic purposes. Another study (77) affirmed the usefulness of CD15 and tryptase as markers to differentiate recent antemortem wounds from post-mortem ones; however, they denied the usefulness of FVIIIra as a vitality marker.

Legaz et al. (78) show an increase in fibronectin and cathepsin-D immunoreactivity and a decrease in P-Selectin in skin wounds from marks from suicide hangings with a post-mortem interval of 19–36 h. However, they state that a limitation of their study could be that the samples were not collected at the time of death, which could influence the immunoreactivity of the proteins studied. Another study (79) demonstrated the usefulness of cathepsin-D as a wound vitality marker. However, Montisci et al. (80) found high levels of cathepsin-D in post-mortem lesions compared to vital wounds collected from living subjects, thus ruling out any usefulness of histochemical quantification of this enzyme for the differentiation between vital and post-mortem lesions. Regarding Selectin-P, some authors (81) found no significant differences in the P-selectin immunoreactivity analysis between vital and post-mortem skin wounds, for which they state that P-selectin is not a specific marker of vital lesions. In a previous study (82), these same authors studied the expression of fibronectin and tenascin in 48 vital wounds and ten post-mortem wounds. They observed a lattice staining for fibronectin at the wound edge and in the dermis of 50% of vital samples, compared to 0% of post-mortem samples, while tenascin was negative in all samples. In contrast, the vital bleeding and post-mortem areas showed positivity for fibronectin and tenascin, so they cannot be considered helpful vitality markers.

Legaz Pérez et al. (83) determined that Fe and Zn concentrations were significantly higher in injured skin from suicide-hanging ligation marks than healthy skin. Furthermore, Ca and Zn decreased, while Fe increased with the severity of the neck injury. On the other hand, they observed a high percentage of negative and moderate expression of cathepsin D and selectin P in damaged skin, correlated with a low iron concentration. Based on these data, the study of proteins and metal ions can be helpful in the characterization and differentiation of injured and uninjured skin. Previously, other authors (84) studied the diagnostic value of ions to differentiate antemortem and post-mortem wounds using atomic absorption spectrophotometry. They found higher Fe concentrations in the skin and muscle of antemortem wounds compared to the control, while the K/Na ratio concentrations were significantly reduced in the antemortem wounds compared to the controls. For this reason, the Fe concentration and the K/Na ratio can be helpful for the differentiation of wounds produced before and after death.

Ali (85) found that altered collagen can also be formed after death, when post-mortem injuries occur, probably due to the physical-chemical changes that collagen fibers undergo, so the presence of altered collagen is not necessarily a sign of vitality.

Other authors (86) detected by HPLC an inflammation mediator, LTB_4_, in an antemortem wound but did not detect it in post-mortem samples. Therefore, their results suggest the detection of LTB_4_ as a useful method to distinguish between antemortem and post-mortem wounds.

Bacci et al. (87) found that the number of mast cells stained for TNF-α increased progressively and significantly over time and became significantly different from controls when the time elapsed after injury was more than 15 min. Furthermore, the post-mortem samples had significantly fewer mast cells and fewer TNF-α positive cells than the antemortem and control sample groups.

Another more recent study (88) used multiplex sandwich immunoassay to analyze different cytokines to discriminate between vital and post-mortem wounds. Cytokine levels (IL-1β, IL-4, IL-6, IL-8, IL-10, IL-12p70, IL-13, and TNF-α) were significantly higher in vital wounds than post-mortem, except for IFN - γ and IL-2. IL-8 was the cytokine that showed the best results for wound differentiation.

Kimura et al. (89) investigated autophagy in human and mouse skin wounds using western blotting. They found a marked reduction in LC3-II and an increase in p62 in antemortem wounds, both human and mouse, with a post-infection interval greater than or equal to half an hour, compared to non-injured skin. However, there were no notable changes in LC3-II and p62 levels in post-mortem wounds.

Finally, two studies (90, 91) suggest the usefulness of the markers CD1a and MHC-II (dendritic cells and Langerhans cells) distinguish between vital and post-mortem injuries, as well as to estimate the interval between injury and death.

## Future Challenges in the Characterization of Human Wounds in Forensic Sciences

In this systematic review, the main results were obtained from studies that attempt to relate different biomarkers with the characterization of wounds, both for estimating the age and for the differentiation between vital and post-mortem wounds. Together, these studies evaluate the potential and limitations of the different biomarkers analyzed for their future use as a forensic tool.

In most of the studies analyzed, immunohistochemistry is the primary method of choice, the basis of this method being the immunological reaction between an antigen present in the study tissue and an applied antibody (6). This method serves to detect vitality and wound markers at the protein level. However, in the review, we have also seen more recent studies that analyze the earliest stage of a reaction at the mRNA level. These are morphological methods such as *in situ* hybridization and molecular biology techniques such as RT-qPCR.

MicroRNAs (miRNAs) are promising biomarkers in forensic sciences because of their small size and their value for degraded or complex samples, which are very common in this field (12). In the articles reviewed in this systematic review, only one analyzed miRNAs in forensic samples, which suggests that the analysis of miRNAs in human samples for the study and characterization of vital wounds is novel and requires more research.

Approximately 30 years have passed since the publication of the first research analyzed in this review, but the molecular characterization of vital wounds remains a topical issue. Estimating the age of vital wounds and the differentiation of vital and post-mortem wounds require further investigation of both biomarkers to analyze and new molecular biology techniques that allow the detection of earlier stages of reactions.

## Data Availability Statement

The original contributions presented in the study are included in the article/supplementary material, further inquiries can be directed to the corresponding author/s.

## Author Contributions

ACR and IL participated in designing the review supervising the data generation, analyzing the data, and writing the manuscript. AL, IL, and SB participated in data generation, organization, writing, and manuscript discussion. All authors contributed to the article and approved the submitted version.

## Conflict of Interest

The authors declare that the research was conducted in the absence of any commercial or financial relationships that could be construed as a potential conflict of interest.

## Publisher's Note

All claims expressed in this article are solely those of the authors and do not necessarily represent those of their affiliated organizations, or those of the publisher, the editors and the reviewers. Any product that may be evaluated in this article, or claim that may be made by its manufacturer, is not guaranteed or endorsed by the publisher.
